# Real-Time Traffic Sign Detection and Recognition Method Based on Simplified Gabor Wavelets and CNNs

**DOI:** 10.3390/s18103192

**Published:** 2018-09-21

**Authors:** Faming Shao, Xinqing Wang, Fanjie Meng, Ting Rui, Dong Wang, Jian Tang

**Affiliations:** Department of Mechanical Engineering, College of Field Engineering, Army Engineering University of PLA, Nanjing 210007, China; shaofaming@163.com (F.S.); Beilimeng1992@163.com (F.M.); rtinguu@163.com (T.R.); dyhkxydfbb@163.com (D.W.); lgdx_tj@163.com (J.T.)

**Keywords:** simplified Gabor wavelets, MSERs, regions of interest, SVM, CNN

## Abstract

Traffic sign detection and recognition plays an important role in expert systems, such as traffic assistance driving systems and automatic driving systems. It instantly assists drivers or automatic driving systems in detecting and recognizing traffic signs effectively. In this paper, a novel approach for real-time traffic sign detection and recognition in a real traffic situation was proposed. First, the images of the road scene were converted to grayscale images, and then we filtered the grayscale images with simplified Gabor wavelets (SGW), where the parameters were optimized. The edges of the traffic signs were strengthened, which was helpful for the next stage of the process. Second, we extracted the region of interest using the maximally stable extremal regions algorithm and classified the superclass of traffic signs using the support vector machine (SVM). Finally, we used convolution neural networks with input by simplified Gabor feature maps, where the parameters were the same as the detection stage, to classify the traffic signs into their subclasses. The experimental results based on Chinese and German traffic sign databases showed that the proposed method obtained a comparable performance with the state-of-the-art method, and furthermore, the processing efficiency of the whole process of detection and classification was improved and met the real-time processing demands.

## 1. Introduction

Traffic signs are road facilities that convey, guide, restrict, warn, or instruct information using words or symbols. With the development of automotive intelligent technology, famous car companies, such as Mercedes-Benz, BMW, etc., have actively invested in ADAS (Advanced Driver Assistance System) research. Commercialized ADAS systems not only include Lane Keep Assist Systems, but also include TSR (Traffic Sign Recognition) systems to remind drivers to pay attention to the speed. If drivers and pedestrians do not notice this information, it can lead to the occurrence of traffic accidents. With the increasing demand for the intelligence of vehicles, it is extremely necessary to detect and recognize traffic signs automatically through computer technology. Research in this area began in the 1980s, to solve this problem.

**Figure 1:**
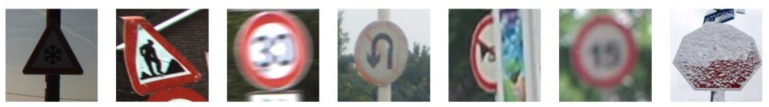
Examples of the difficult situations faced in traffic sign detection and recognition: Undesirable light, disorientation, motion blur, color fade, occlusion, rain, and snow.

To make them easy for drivers to read and recognize, traffic signs are often designed to be of a particular shape and color with symbols inside, so that there is a significant difference between the traffic signs and the background. For example, the speed limit 60 traffic sign is a circular shape with a strong number “60”. These features are also important information for traffic sign recognition systems. However, traffic sign recognition is not an easy task, because there are many adverse factors, such as bad weather, viewpoint variation, physical damage, etc. The difficulties in this area that we faced, were as follows:

- Although the same kind of traffic signs have some consistency in color, in outdoor environments the color of the traffic signs is greatly influenced by illumination and light direction. Therefore, the color information is not fully reliable.
- As vehicle mounted cameras are not always perpendicular to the traffic signs, and the shape of traffic signs are often distorted in road scenes, the shape information of traffic signs is no longer fully reliable.
- Traffic signs in some road scenes are often obscured by buildings, trees, and other vehicles; therefore, we needed to recognize the traffic signs with incomplete information.
- Traffic sign discoloration, traffic sign damage, rain, snow, fog, and other problems, are also given as challenges in the process of traffic sign detection and classification.
- Some challenging examples are shown in Figure 1.

In this paper, we present a real-time traffic sign recognition system consisting of traffic sign detection and classification. The main contributions of the paper are outlined as follows:

- We propose simplified Gabor filters to preprocess the grayscale images of traffic scenes, to enhance the edges and strengthen the shape information. In addition, this could make the non-edge areas of painted artificial objects, such as traffic signs, more stable and reduce the noise in such areas.
- We use the maximally stable extremal regions (MSERs) algorithm to process the simplified Gabor filtered map to find the regions of interest more effectively, and we used our defined rules to filter out significant negative samples.
- We first used an eight-channel simplified Gabor feature as the input of the CNNs, which were defined as a pre-convolution layer of the convolutional neural networks (CNNs) for traffic sign classification.
- Our method performs only one feature extraction through the detection and classification stage, which causes feature sharing throughout the two stages. Compared with algorithms used in the different feature extraction methods, in the detection and classification stage, this saves a lot of processing time and makes it feasible for use in real time applications.

The rest of our paper is organized as follows: In Section 2, we introduce the related work. In Section 3, we provide an overview of the method. The details of our traffic sign detection method are presented in Section 4. Section 5 describes the traffic sign classification method, and an experimental evaluation is provided in Section 6. Finally, we present some conclusions and future work.

## 2. Related Works

In general, traffic sign recognition mainly includes two stages: the first stage is traffic sign detection, which concerns the location and size of the traffic signs in the traffic scene images, and the second stage of the process is traffic sign recognition, which pays close attention to the classification of what exact class the traffic signs belong to. Traffic sign detection is usually based on the shape and color attributes of traffic signs, and traffic sign recognition is often used with classifiers, such as convolutional neural networks (CNNs) and SVM with discriminative features.

Traffic signs have a strict color scheme, which includes red, blue, and white that allow us to distinguish traffic signs from the background scene. It is not difficult for human beings to distinguish traffic signs from a background, so for a computer detection system, color information is also an important feature. In the present research, color-based traffic sign detection method is shown to be the most straightforward and simplest method [1,2,3,4]. As red, green, blue image (RGB) is too sensitive to illumination, color space conversion algorithms have often been applied to traffic sign detection. For example, Shadeed et al. [5] segmented the image by the U and V chromaticity channels in YUV space, where U is positive and V is the color red. This information was combined with the hue channel of the hue, saturation, value (HSV) color space to segment the red road signs from the image. In Reference [6], thresholds were used in the color channel of the HSV color space, to segment the red road signs. Unfortunately, due to strong light, poor light, and other bad weather conditions, color-based detection methods often fail to achieve better results.

Except for countries like Japan and a few others, traffic signs have pairs of different signs when converted to grayscale which appear the same, and traffic signs from most countries have a strict shape and symbol distinction. This design allows color blind and color weak drivers and pedestrians to recognize traffic signs without or with less color information. Based on such features, some algorithms completely ignore color information and adopt shape-based image segmentation [7,8,9]. In addition to the above-mentioned factors, some algorithms use image features other than the raw color space and shape information. The authors in Reference [10] used the color probability model, and traffic sign proposals were then extracted by finding the most stable extremal regions on these maps. Reference [11] used rotation invariant binary pattern-based features. In any case, finding good feature expression is an important part of traffic sign detection, and is also an important research topic in this field.

Recently, as deep learning methods have demonstrated prominent representation capacity and achieved outstanding performance in traffic sign recognition, more scholars have applied technologies to this area [12,13,14,15]. Jin [12] used a convolution neural network based on deep learning with a hinge loss stochastic gradient descent method, which achieved a high recognition rate. Dan [13] provided a multi-column deep neural network for traffic classification running on a graphic processing unit (GPU), and obtained a better-than-human recognition rate. Qian [14] used CNN as the classifier to learn the discriminative feature of max pooling positions to classify traffic signs and obtained a comparable performance with the-state-of-the-art method. Ali [15] used a procedure based on color segmentation, histogram of oriented gradients, and CNN, for the detection and recognition of traffic signs, which achieved better classification accuracy and computational speed. However, in the field of traffic sign recognition by deep learning methods, to meet the demands of real time application, it is necessary to further study the choice of discriminative features and to research the network structure to improve classification accuracy and processing time.

For a long time, there were no public and challenging datasets available in this area, but this situation changed in 2011. Larsson and Felsberg [16] and Stallkamp et al. [17] introduced challenging databases, including annotations for traffic sign detection classification. These databases included the Belgium Traffic Sign Classification (BTSC), the German traffic sign detection benchmark (GTSDB), and the German Traffic Sign Recognition Benchmark (GTSRB) databases. In particular, the GTSDB and GTRSB have attracted more scholars to find new methods to verify through this database, and some of them have achieved good results. The existence of open databases has made the research methods comparable. The above traffic signs databases belong to European traffic laws and regulations. Some Chinese scholars have paid more attention to the problem of traffic sign detection and recognition in the Chinese traffic environment [18,19,20,21]. The construction of a traffic sign database for Chinese traffic environment is very necessary. Fortunately, the Chinese Traffic Sign Dataset (CTSD) was constructed by Yang [10], which has attracted the attention of more Chinese researchers.

## 3. Overview of Our Method

**Figure 2:**
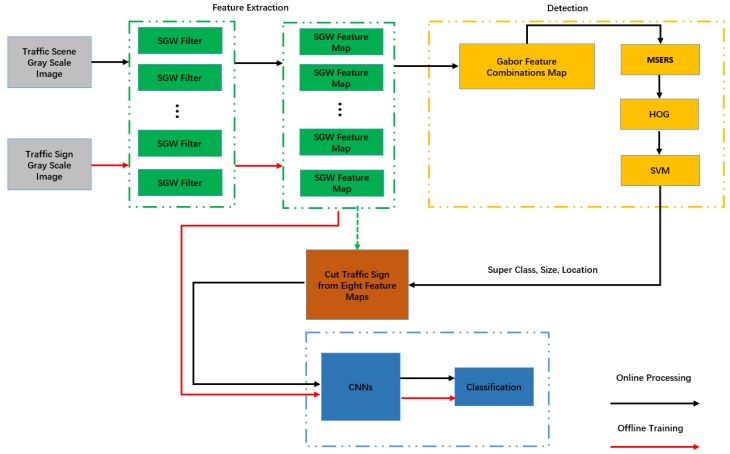
The pipelines of our method.

The processing flow of our traffic sign detection and recognition system is illustrated in Figure 2. The red line with the arrow indicates the offline training flow of the subclass classified network, and the blue line with the arrow indicates the whole process of online detection and classification. In detail, during the feature extraction, we filtered the road scene grayscale images by four orientations, and two scale simplified Gabor filters, where its parameters were optimized by experiments. The simplified Gabor filters were used to enhance the edges and smooth the non-edge areas of the images. Then, the eight output feature maps were synthesized into one map by maximizing the value at each pixel. In the synthesized Gabor feature map, the edges of the traffic signs were strengthened and the areas inside the traffic signs were smoothed. The feature extraction process is shown in the green area of this figure. During the detection stage, we first used the maximally stable extremal regions (MSERs) algorithm to obtain the regions of interest (ROI) and filtered out the areas where there was little possibility for existence of traffic signs, according to our defined filter rules. Then, the proposed regions were classified by SVM to get the super-class of the traffic signs which each area belonged to. The detection process is shown in the yellow area of the figure. Finally, we classified the subclasses of the traffic signs using CNN, which is shown in the blue area of the figure. The input of the CNN was eight simplified Gabor feature maps, which were convoluted to traffic signs by the SGW. What needs to be stressed is that in the real time processing stage, the input of the CNNs was the matrix that was cut from the eight simplified Gabor feature maps in the detection stage. The matrix corresponded with the area that was detected as a traffic sign at the same stage. In the red line flow part of Figure 2, we trained the CNN with eight simplified Gabor feature maps, convoluted from the traffic signs of GTSRB.

## 4. Traffic Sign Detection

Traffic sign detection, as one category of object detection, is attracting the attention of many researchers. Compared with general object detection, traffic signs are designed with a strict color and shape, so that they can be distinguished more easily from the background through these characteristics, either by a human being or an intelligent machine. Therefore, the detection method for traffic signs is generally based on color, shape, or both. However, the color images captured by mounted cameras on a vehicle often fail to highlight the shape information, and cannot express the color information stably, which causes the loss of such information. In existing technology, color information enhancement [22] or shape information enhancement [3] methods are usually used as the preprocess stage of traffic sign detection. The shape is determined by the edges of the objects in the images, so strengthening the information of the edges of the object and weakening the non-edge area can enhance the shape information. In our method, we enhanced the shape information of traffic signs by edge enhancing technology through simplified Gabor filters.

### 4.1. Simplified Gabor Wavelet Model

The human visual system is composed of filter banks. The Gabor functions, with different orientations and scales, can model the responses of the respective filters of human beings. Gabor features have been found to be particularly effective for texture identification and representation [23,24]. This characteristic of the Gabor filter has been applied to object detection [25], fingerprint recognition, facial expression recognition [26], image segmentation [27], etc.

In the spatial domain, a 2D Gabor filter is a Gaussian kernel function modulated by a sinusoidal plane wave, where the function is shown by Equation (1):

(1) $$G\left(x,y\right)=\exp\left[-\frac{\left(x^{2}+y^{2}\right)}{2\sigma^{2}}\right]\cdot \exp\left[j\omega \left(x\cos\theta +y\sin\theta \right)\right]\;$$

where σ is the standard deviation of the Gauss function in the x- and y-orientations; and ω denotes the spatial frequency. To convolute the traffic scene with the Gabor filter with an input image, I(x,y), the output of the Gabor filtered map, ϕ(x,y), is given as Equation (2). Operator ⊗ in the function denotes the 2D convolution operation.

(2) ϕ(x,y)=G(x,y)⊗I(x,y) 

Pellegrino et al. [28] proposed that the imaginary part of the Gabor filter was an effective and robust edge detection method. The imaginary part of GW is shown in Equation (3), and σ and ω satisfy the condition σ⋅ω≤1:

(3) $$G\left(x,y\right)=\exp\left[-\frac{\left(x^{2}+y^{2}\right)}{2\sigma^{2}}\right]\cdot \sin\left[\omega \left(x\cos\theta +y\sin\theta \right)\right]\;$$

The Traditional Gabor wavelet (TGW) was introduced to enhance the edge of the object of scenes for many years [29,30], as it produces a better segmentation effect for this area. However, the edges of objects in images such as traffic signs, always had random orientations. To get the direction of the Gabor filter close to the edge, it would need filters of many different TGW orientations. The more filters with different orientations and scales, the more completely the information is expressed. From another point of view, the more the filters present, the more computational time required. One of the most serious problems in multi-orientations and multi-scale Gabor filter applications is time consumption. This is the main reason that TGW might be impractical for real-time application of traffic sign detection and recognition. Therefore, the reduction of processing time is one of the key problems in this area.

To reduce time consumption in filtering and improve computational efficiency, we used a set of simplified Gabor filters to extract the features for edge detection, as proposed in Reference [31]. The SGWs were in the form of simple patterns for different scales and orientations, which could be computed efficiently. As proposed in Reference [31], these SGW features could be computed for each pixel position without requiring the use of Fast Fourier Transform (FFT), which greatly saved time.

The selected quantization levels for an SGW, were the same as those in Reference [30]. We set one quantization level of the SGW to zero. As the image part of the Gabor function is antisymmetric, the positive and negative quantization level values were the same and we denoted them as *n_l_*, so that there were 2*n_l_*+ 1 quantization levels in our approach. To discretize the Gabor amplitude, supposing that the largest magnitude of the TGW was *A* and *k* = 1, …, *n_l_*, the quantization levels *q_positive_* and *q_negtive_* were defined by Equations (4) and (5), respectively. Then, the traffic sign scene was convolved with the SGW kernels with different scales and orientations:

(4) $$q_{positive}=\frac{A}{2n_{l}+1}\cdot 2k\;$$

(5) $$q_{negtive}=-\frac{A}{2n_{l}+1}\cdot 2k\;$$

As a trade-off between speed and efficiency, we chose four orientations and two scale filters. The output of convoluting image I(x,y) with a SGW was denoted as $\phi_{\omega_{i},\theta_{j}}^{\prime }\left(x,y\right)$. We synthesized the eight filtering maps into one map, denoted as $\phi_{\omega ,\theta }^{\prime \prime }\left(x,y\right)$, by finding the maximum value of the eight filtered map at each pixel with Equation (6), where i=0,1 and j=0,1,2,3:

(6) $$\phi_{\omega ,\theta }^{\prime \prime }\left(x,y\right)=\max\left\{\phi_{\omega_{i},\theta_{j}}^{\prime }\left(x,y\right),\;i=0,1\;\mathrm{and}\;j=0,1,2,3\right\}\;$$

Orientation selection involved adopting an equal half plane angle selection method, which meant that $\theta_{j}$ belonged to (0,π) instead of (0, 2π), because the filters’ cores had the same edge extraction capacity in the opposite direction, which allowed the filtering time to be reduced to half of the original. As the direction of the simplified Gabor kernel was divided into the two-dimensional plane in the same step, it balanced every orientation and robustness to several factors, such as translation and rotation. This algorithm is invariant to translation, because it does not depend on the position of the appearance of the blob in the scene. The output feature $\phi_{\omega_{i},\theta_{j}}^{\prime }\left(x,y\right)$ is the result of convoluting the image (I(x,y)) with the simplified Gabor filter, and its pattern is dependent on the two parameters: θ and ω. As the edges are the local feature in the image, the purpose of convolution is to extract the local features of the image, so the size of the convolution kernel should be sufficiently small. In our approach, the best window size was 5 × 5 throughout the experiments.

The purpose of simplified Gabor preprocessing was to strengthen the edge, but there are many algorithms for edge enhancement, such as the Canny algorithm and the TGW algorithm with specific parameters, etc. In the following text, we analyze and compare the computational complexities of the three different edge-detection algorithms: The Canny algorithm, TGWs, and SGW. In our context, the computation of an algorithm refers to the number of real additions and real multiplications required for edge detection. The number of additions does not count the operations required for generating the pixel coordinates in our analysis. As FFT was employed for TGW-based edge detection, we assumed that the image size was a power of two. For the SGW-based and the Canny algorithms, the image could be any size. The SGW features at any pixel position were able to be computed individually and efficiently.

From Table 1, it is seen that the SGW-based approach achieved the best computational effect, compared to the Canny and traditional Gabor wavelet methods. The orientation number and scale of the traditional Gabor wavelet and simplified Gabor wavelet methods were two and four, respectively. Although the SGW was a little faster than the Canny, the output of the Canny algorithm was a binary image, which would cause a lot of image information loss, and could not ensure sufficient detection and classification performances.

**Figure 3:**
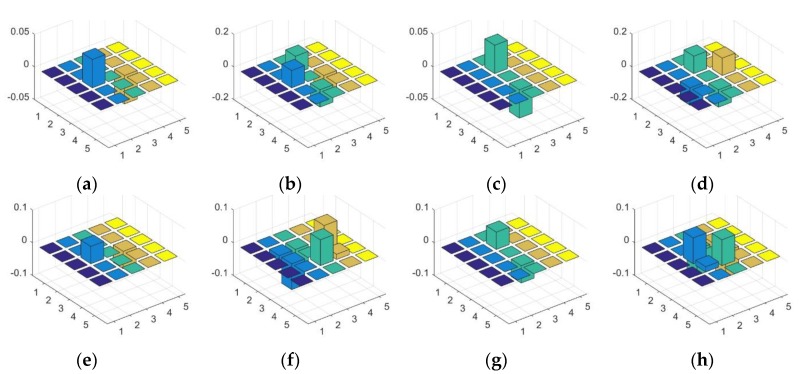
The eight SGW filters shown in 3D histograms with the following parameters: (**a**) ω=0.3π θ=0; (**b**) *ω* = 0.3π, *θ* = π*j*/4; (**c**) *ω* = 0.3π, *θ* = π*j*/2; (**d**) *ω* = 0.3π, *θ* = 3π*j*/4; (**e**) ω=0.5, π θ=0; (**f**)  ω=0.5π, θ=πj/4; (**g**) ω=0.5π, θ=πj/2; and (**h**)  ω=0.5π, θ=3πj/4.

**Figure 4:**
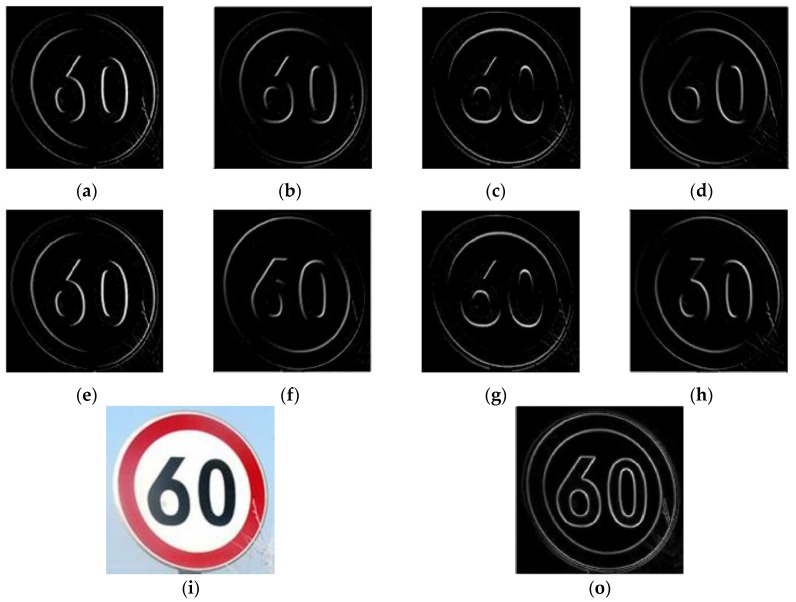
Sample processing by eight SGW filters and output of maximization at each pixel in the eight feature maps: (**a**) *ω* = 0.3π, *θ* = 0; (**b**) *ω* = 0.3π, *θ* = π*j*/4; (**c**) *ω* = 0.3π, *θ* = π*j*/2; (**d**) *ω* = 0.3π, *θ* = 3π*j*/4; (**e**) ω=0.5π, θ=0 ; (**f**) ω=0.5π, θ=πj/4; (**g**) ω=0.5π, θ=πj/2; (**h**)  ω=0.5π, θ=3πj/4; (**i**) the input of the image; (**o**) the output of the synthesized map.

The eight SGW filter kernels are shown in Figure 3. The first and second rows of Figure 4 show the example of the speed limit 60 sign, convoluted by SGW with the eight SGW filter kernels. The image (o) in the third row shows the result of synthesizing the eight filtering maps into one map by finding the maximum value of eight filtered maps at each pixel with Equation (6). As shown in image (a) to image (h) in Figure 4, the orientation of the edge of the traffic sign that coincided with the SGW filter was obviously strengthened. Furthermore, all the traffic sign edges of the synthesized filtering maps are strengthened in the last image of Figure 4. From the above, we concluded that SGW could really enhance the edges of the images, whilst saving computing time.

**Figure 5:**
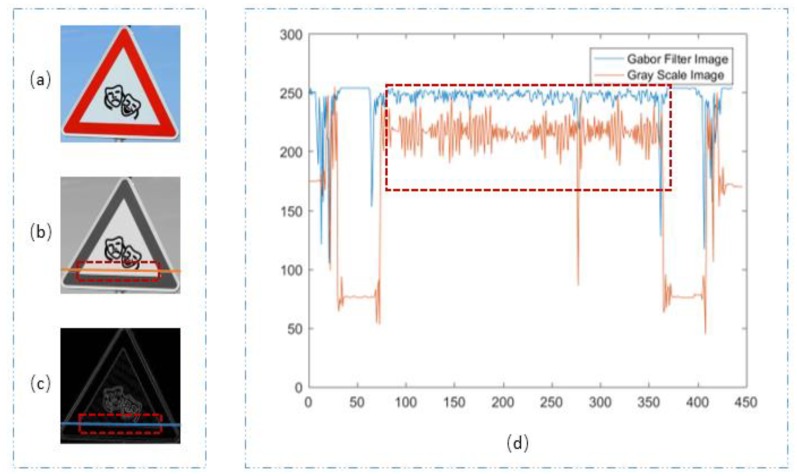
(**a**) Traffic sign of RGB; (**b**) grayscale image; (**c**) synthetic Gabor filtered map; (**d**) two vector contrast graphs.

**Figure 6:**
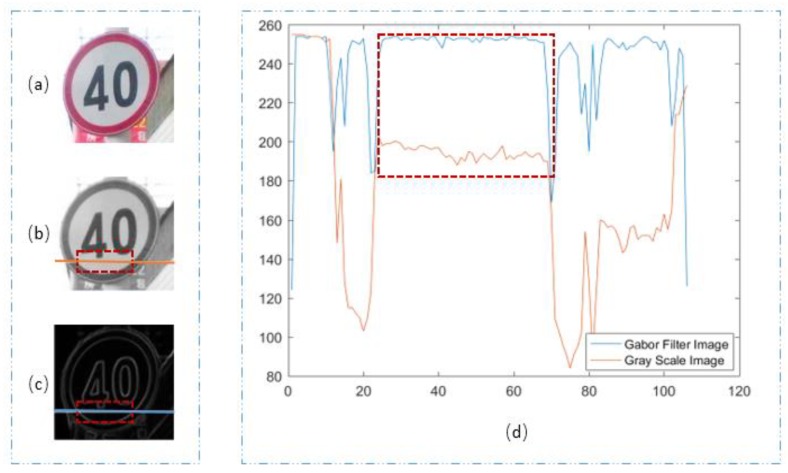
(**a**) Traffic sign of RGB; (**b**) grayscale image; (**c**) synthetic Gabor filtered map; (**d**) two vector contrast graphs.

Through the above analysis, we can clearly see that SGW had better edge enhancement capacity. In the following section, we analyze the filtering effect of SGW for the non-edge regions. The images (a) in Figure 5 and Figure 6 are the RGB images of the traffic scene. The images (b) in Figure 5 and Figure 6 are grayscale images which were converted from the RGB images. The images (c) in Figure 5 and Figure 6 are the synthetic maps. We took one row vector from each of the grayscale images (b) in Figure 5 and Figure 6, as shown in the orange line at the same position, in each of the images (c) in Figure 5 and Figure 6, we took one row vector as shown in the blue line. To make the two vectors visually comparable, the vector from the synthetic maps was transformed by Equation (7). $V_{1}\left(i\right)$ denotes the vector taken as above, *L* is the length of the vector $V_{1}\left(i\right)$, and $V_{2}\left(x\right)$ is the vector after transformation:

(7) $$V_{2}\left(x\right)=255-\frac{V_{1}\left(x\right)}{\max\left[\left|V_{1}\left(i\right)\right|\right]}\times 255,\;x=1,\ldots ,\;L\;i=\;1,\ldots ,\;L\;$$

We compared the two vectors in the images (d) of Figure 5 and Figure 6. In the two images, the orange curves represents the vectors obtained from the grayscale map, and the blue curves represents the vectors from the synthesized map. The areas in the red dashed line box corresponds to the areas of the vectors of images (images (b) of Figure 5 and Figure 6, images (c) of Figure 5 and Figure 6) in the same style box. The vector cut by the red dashed line box was the painted area in the traffic sign, which is more uniform when compared with the nature object in the traffic sign scene. In the two figures, we can see that the amplitude of the blue lines in the curves are smaller than that of the orange ones, which means that the noise of the painted image inside the traffic sign is suppressed, and the image was smoothed. In image (d) of Figure 6, the orange curve from the vector of the grayscale map was in a state of decline. The reason for the decline of the vector was an imbalance in illumination, as we could see from the image that the traffic sign was slanted towards the cameras. In image (d) of Figure 6, when compared with the orange curve, the blue curve is balanced, which means that the imbalance illumination was rectified by the SGW filters. Through many experiments, we found that that SGW could not only enhance the edges, but could also smooth the surface of artificial objects, such as painted non-edge areas. This meant that the SGW could reduce noise in the areas of the surface of artificial objects, such as painted areas where the color was uniform. Furthermore, the SGW could also overcome the effect of imbalance illumination. These characteristics of SGW are very important for the next stage of our method.

### 4.2. Traffic Sign Proposal Extraction

To detect the traffic signs in the traffic scene, in the following stage, we aimed to find the traffic sign proposal regions proposed by the MSERs region detector. The MSERs algorithm has often been used as a method for blob detection in images. This technique was proposed by Reference [32] to solve problems regarding the robust wide-baseline stereo, and was first used for traffic sign detection in Reference [33]. MSERs are regions that maintain their shape, whilst thresholding the image at several levels. The advantage of this approach is its robustness to variations in contrast and lighting conditions in outdoor traffic scenes. This method has been widely used in natural scene text location and traffic sign detection. Reference [33] used MSERs to detect traffic signs on a grayscale map. In Reference [10], MSERs were used to detect traffic signs with a region proposal from the color probability map, which increased the contrast between the target and background. Unlike References [10,33], we extracted the maximally stable extremal regions from the synthetic simplified Gabor filtered map.

To find the MSERs, the value of each pixel in the synthetic simplified Gabor filtered map was set to 1 if its intensity was larger than a threshold; otherwise, it was set to 0. When the SGW map was threshold at several levels, the most stable connected components that maintained their shapes were defined as MSERs. As mentioned above, after being filtered by SGW, the edges of the traffic signs were strengthened and the area inside the traffic sign was smoothed. According to the algorithm principles of MSERs, this could help the regional proposal of traffic signs to be obtained.

**Figure 7:**
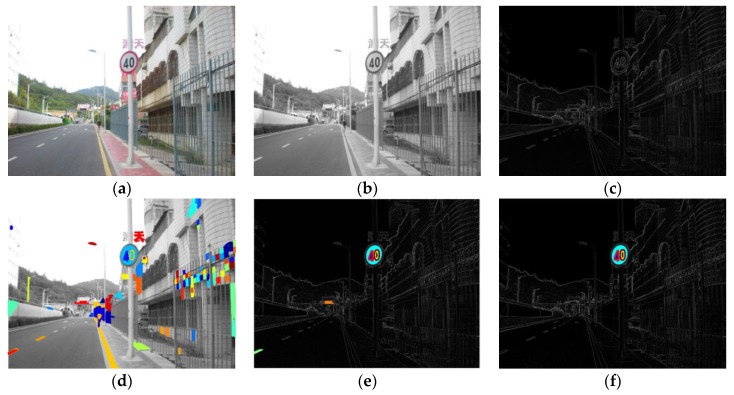
(**a**) Original scene RGB image; (**b**) grayscale image; (**c**) synthetic SGW filtered map; (**d**) maximally stable extremal regions (MSERs) on grayscale map; (**e**) MSERs on synthetic SGW filtered map; and (**f**) segmentation results after taking account the filter rules.

The background of natural scenes is complex, and the MSERs detector will detect a large number of areas which are not traffic signs, as shown in image (d) and image (e) of Figure 7. It is necessary to design filter rules to reduce the number of candidates. From this approach, the processing time of the system can be greatly reduced, and accuracy can be improved. In our methods, the features used to filter out the candidates included the following: The minimum and maximum height of the proposed areas, the minimum and maximum width of the proposed areas, the aspect ratio, and the duty ratio. The aspect ratio was defined as the width of the proposed area divided by the height of the proposed area. The duty ratio was defined as the MSERs area divided by the bounding box area. The bounding box was the smallest external rectangle of the traffic sign. The calculation of the duty ratio is shown as Equation (8). When the pixel I(x,y) was in the MSERs, the value of f(x,y) was 1; otherwise, it was 0. The width and height in the formula refer to the width and height of the bounding box, respectively. The numerical ranges of these parameters in the filter rules are shown in Table 2. The numerical ranges of the parameters in Table 2, were selected by empirical data in the experiment.

(8) $$M_{B}=\frac{\sum_{x=1}^{width}\sum_{y=1}^{height}f\left(x,y\right)}{width\times height}\;$$

Figure 7 shows examples of the traffic scene images processed by SGW and MSERs, filtered by the rules we designed. Image (a) and image (b) of Figure 7 are the RGB and grayscale images of the traffic scene. Image (c) of Figure 7 is the synthesized SGW map, with the maximum values of the eight filtered map at each pixel. From image (c) of Figure 7, we can clearly see that the edges and the inside symbol of the traffic signs were enhanced. Image (d) and image (e) of Figure 7 are the grayscale image and synthesized SGW map after being processed by MSERs. This example shows that the number of the maximally stable extremal regions from the synthesized SGW map was obviously less than that of the grayscale image; however, these regions contained the traffic signs areas. Image (f) of Figure 7 shows the regions left after being filtered by the rules defined above.

### 4.3. Traffic Sign Detection

Through preliminary filtering of the MSERs by the filter rules, we were able to greatly reduce the number of proposed traffic sign areas. In other words, the number of candidate areas was reduced without reducing the recall rate. From the point of view of processing time, the decline in the number of the proposed regions meant saving time in the next stage. In addition, it avoided the multi-scale sliding window detection method, which is time consuming and has low efficiency. As a basic demand for traffic sign detection, a more detailed classification algorithm must be used to divide candidate regions into their super classes.

To obtain the super classes that the proposed areas should belong to, we first extracted the Histogram of oriented gradient (HOG) features from the SGW feature map, which we named the SGW-HOG features, and then used a multiclass SVM classifier trained with SGW-HOG features. As there were two super classes in the traffic signs, we trained a three-class SVM as there was a third super class of negative samples.

**Figure 8:**
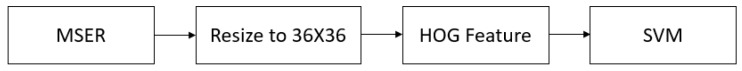
The processing operation for super class classification.

In the process of transforming the SGW map to a HOG feature map, we did not use the method of transforming the whole map to the HOG feature map, but only transformed the candidate proposed regions. In this way, processing time was greatly saved. The processing operation is shown in Figure 8.

The HOG feature is characterized by the gradient direction information of the local region of the image as the representation of the local image region; the process of HOG feature extraction based on SGW features is as follows:

- The normalized sample image is used as the input, and the gradients in the horizontal and vertical orientations are calculated by the gradient operator.
- Statistic local image gradient information, where the sample image is divided into several pixels (cell), and the gradient direction is divided into nine intervals (bins). In each unit, the gradient direction of all pixels is counted in the direction interval, and a nine-dimension eigenvector is obtained.
- The adjacent four units form a block, so the characteristic vectors of all the elements in a block are connected in series to obtain the feature description vectors of the block region (36 dimensions).
- The sample image is scanned in blocks, and the scanning step is the cell. Finally, all the features of the block are combined to get the feature of the sample.

In the detection stage, we obtained information on the location and the size of the traffic signs in the traffic scene. Furthermore, we determined the super classes of traffic signs, to deduce whether the traffic sign was a circular or triangle one. After this work, we were able to classify the traffic signs of the two super classes into their subclasses independently.

## 5. Traffic Sign Classification

The convolutional neural network (CNN) is a type of multi-layer neural network, which extracts features by combining convolution, pooling, and activation layers. The CNN is widely used in the field of pattern recognition. Many researchers have applied the CNN to traffic sign recognition and detection and have achieved good results. The CNN achieved perfect performances in the 2011 International Joint Conference on Neural Networks (IJCNN) [34,35]. Recently, the CNN has been adopted in object recognition with high accuracy. Most of these models use raw images rather than hand-coded features, and most of them regard feature extraction and classification as a whole; this is known as end to end classification. Although raw image-based CNN image classification methods have achieved better performance, many scholars have also further researched the performance of CNN classification after feature transformation. The rotation invariant binary pattern-based feature, was used as the input for an artificial neural network in Reference [11]. Grey relational analysis was used before traffic sign recognition in Reference [36], and distance to border feature express was used as the input of the CNN in Reference [37]. Research into the expression of image features has attracted significant attention in the field of image classification.

The Gabor filter attempts to imitate the characteristics of certain visual cells of mammals and has been successfully used as a preprocessor and feature extractor for a large variety of image processing applications, such as pattern recognition and analysis motion. In image classification fields, the Gabor feature has been widely used as the input of classifiers. In References [29,37,38], the Gabor feature was used for face recognition, mostly obtaining the performance of state-of-the-art methods. Many researchers have used the Gabor feature as an input of CNN and achieved better results. In the fingerprint image recognition area, Reference [39] used the Gabor filter to preprocess the raw image and made it the input of the CNN. In Reference [40], the speech signal was filtered by the Gabor filter, and then recognized by the CNN. In Reference [36], the Gabor filter was used as the first layer of the neural network and experiments regarding the open database in the area of face detection, digit recognition, and objection recognition, where it obtained a comparable performance with energy efficiency and fast learning during the training of the CNN. Therefore, the use of the special parameters of Gabor-based preprocessed images as the input of the CNN for image classification is very valuable research.

In the detection stage of our method, we classified the traffic signs into super classes. We obtained information about the locations and sizes of the traffic signs in the traffic scene, and whether the traffic signs were circular or triangular signs. However, it is not known which subclass these traffic signs belonged to. In traffic sign design in China and Germany, there are no two traffic signs with the same shape and symbol but different colors; therefore, the traffic signs can be classified just by shape and the inside symbol. Moreover, it is also mentioned in Reference [41] that color information has little effect on the classification of traffic signs. As mentioned above, simplified Gabor filters can enhance the edge information of the image. This ability of the SGW can be used to strengthen the edges and internal symbols of traffic signs, which is conducive to shape-based classification. In our method, to classify the traffic signs, we used the simplified Gabor feature map as input, followed by the CNN.

We designed two CNNs to classify the subclasses for the two super classes of traffic signs independently. One CNN was used to classify the subclass of circular traffic signs, and the other was used for the triangular traffic signs. The CNN for circular traffic signs classified the super class into 20 subclasses, and the CNN for triangular traffic signs classified the super class into 15 subclasses. We believe that further partitioning the subset of the database and classifying it independently, reduces the interference between different classes and improves classification accuracy. To verify the effectiveness of this approach, we designed the third CNN that classified all the traffic signs.

**Figure 9:**
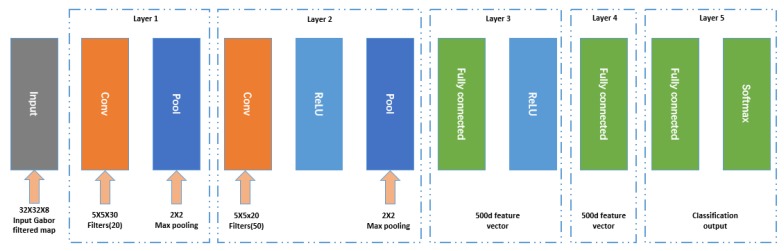
The structure of the three convolutional neural network (CNNs).

We used four orientations and two scales for the SGW filtered image, as the eight-channel input of the three CNNs. The parameters of SGW were the same as that of the detection phase. When our algorithm was used in online applications, the features used in the classification phase were the features extracted from the detection phase, and features extracted twice during detection and classification were avoided. In this way, time was greatly saved, and the method better met the demands of real-time. As the inputs of the CNN must all be the same size, we resized all the images to a size of 32×32 before being filtered by the SGW. The three CNNs had the same structure, which is shown in Figure 9. The architectures of the three CNNs were almost the same as the expected number of output classes.

## 6. Experimental Results

In this section, we first give a brief introduction to the database of German and Chinese traffic scenes. Then, we evaluated the accuracy and computing time consumption of the traffic sign detection and classification through experiments. Finally, we analyzed the reasons that caused false results in some of the test samples.

### 6.1. Experimental Dataset and Computer Environment

**Figure 10:**
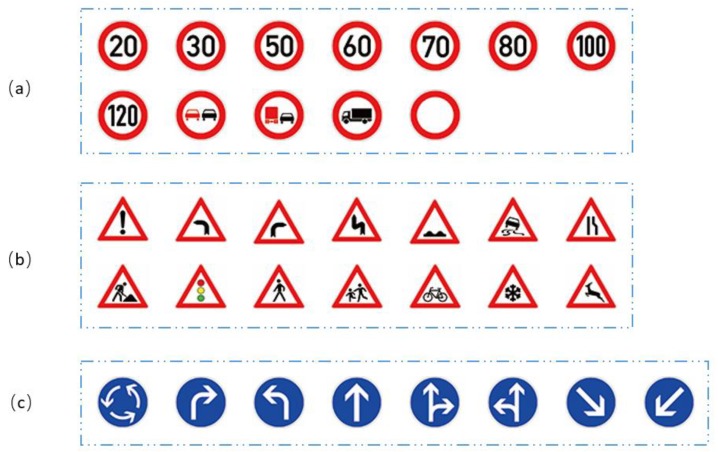
Examples of the subclasses of the German traffic sign detection benchmark (GTSDB): (**a**) prohibitory traffic signs; (**b**) danger traffic signs; (**c**) mandatory traffic signs.

**Figure 11:**
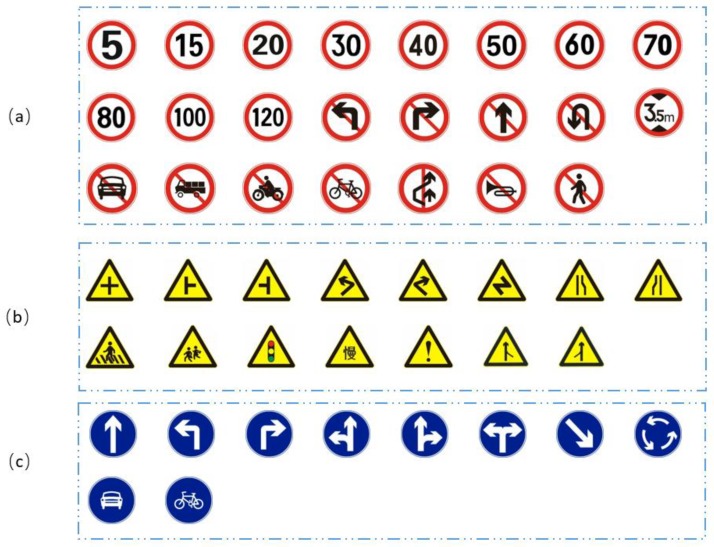
Examples of the subclasses of the Chinese Traffic Sign Dataset (CTSD): (**a**) prohibitory traffic signs; (**b**) danger traffic signs; (**c**) mandatory traffic signs.

In the detection stage, the publicly available German traffic sign detection benchmark (GTSDB) dataset and the Chinese traffic sign dataset (CTSD) were adopted for performance evaluation. In both the GTSDB and CTSD, there are three classes of traffic signs: Prohibitory, mandatory and danger, and the shapes of the traffic signs are circular and triangle. Figure 10 shows the examples of the subclasses of GTSDB, and Figure 11 shows the examples of subclasses of CTSD.

The GTSDB dataset included 900 high-resolution natural scene images of the road environment in Germany. The sizes of those images were 1360 × 800 pixels, and the size of the traffic signs varied from 16 × 16 pixels to 128 × 128 pixels. These 900 pictures were divided into two parts: 600 of which were used for training, and 300 were used for testing. There were 1213 traffic signs in the GTSDB scene pictures, and zero to six traffic signs in one traffic scene. In the test dataset of GTSDB, there were 161 prohibitory traffic signs, 63 danger signs, and 49 mandatory signs, respectively.

For research into Chinese traffic sign detection, we used the Chinese Traffic Sign Dataset (CTSD) from Reference [10]. In the CTSD, there were 1100 images, which included 400 for testing and 700 for training. The sizes of the images were 1024 × 768 or 1280 × 720. The traffic signs included prohibitory traffic signs with a circular shape and red color, danger traffic signs with triangular shape and yellow color, and mandatory traffic signs with a circular shape and blue color. In the CTSD test dataset, there were 264 prohibitory traffic signs, 129 danger signs, and 139 mandatory signs, respectively.

Our research into traffic sign recognition was based on the Germany traffic sign recognition benchmark (GTSRB), and the sizes of the traffic signs varied from 15 × 15 pixels to 250 × 250 pixels. The total number of traffic signs in GTSRB was 51,839: 12,630 images for testing and 39,209 images for training. Consistent with the GTSDB, the traffic signs of GTSRB were divided into three main categories, including 43 subclasses.

Traffic sign detection and recognition algorithms were conducted in a Matlab development environment. Additionally, the hardware configuration of the system was Intel^®^ Core^TM^ i5-6300U CPU@2.40 GHz 2.50 GHz, 8 GB DDR3, Windows 10 64-bit, and Intel^®^ HD Graphics 520 Graphics.

### 6.2. Traffic Sign Detection

To evaluate the performance of our region proposal method, we used the recall rate and false negative (FN) to analyze the performance of our algorithm. The recall rate was defined by Equation (9), and FN refers to the positive samples with a negative predictive value:

(9) $$Recall=\frac{true\;\;positives\;\;detected}{total\;\;true\;positives}\times 100\%\;$$

Table 3 illuminates the traffic proposal extraction results on the GTSDB and CTSD test sets. As we can see from the table, with the MSERs method on the SGW map, there were only one and two traffic signs that had not been proposed in the GTSDB and CTSD, respectively. The traffic sign proposal ability of our method was better than that in Reference [10], and the method of MSERs on the gray-scale image. After image preprocessing by the SGW, the average number of proposals was significantly lower than the other two methods. What needs to be mentioned is that using the defined filter rules, the average number of proposals was further reduced without a decline in the recall rate. A high recall rate and a small number of traffic sign proposals were very important for the fast and high accuracy of the super class classification of the next phase.

In this stage, we used SVM to classify the shape that the ROIs belonged to and identified the super-class of the candidate area and tried to classify the shape of the areas segmented from the SGW map and filtered by the defined filter rules from Table 2.

First, we focused on the size of the training samples and the selection of HOG parameters. To verify the classification effect under different parameters, the dataset was normalized to five different sizes. The HOG specific parameters of the five different sizes are shown in Table 4. The training samples of different sizes were extracted according to their respective parameters, and then sent into the linear SVM training to obtain the detection classifier, where the selection of parameters was the same.

The five HOG features were used to train the classifier, to test the performance on the GTSDB and CTSD datasets, as shown in Table 3. From Table 4 and Table 5, we can draw the conclusion that an increase in the sample size and feature dimensions increased the training time. Although HOG1, HOG4, and HOG5, had faster detection times than HOG2, the detection accuracy of HOG2 was higher than the other four HOG features. To ensure a high detection rate, HOG2 was chosen as the feature of detection.

**Figure 12:**
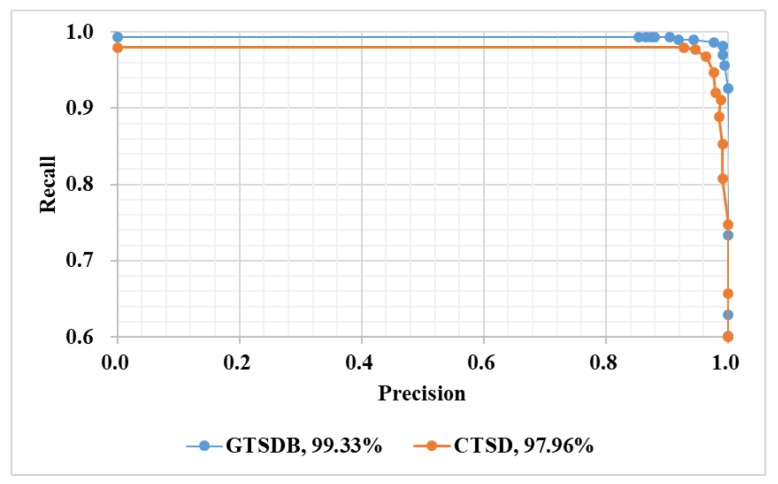
Detection precision recall curves on the GTSDB and CTSD. The y-coordinate was adjusted for clarity.

The two precision recall curves from the datasets of GSTDB and CTSD are shown in Figure 12. Precision was defined by Equation (10). As shown in the figure, with the same precision, the recall from GTSDB was better than that from CTSD; in addition, the AUC (area under curve) of the GTSDB was better than that of CTSD, probably because the quality of the CTSD’s pictures were not as good as that of GTSDB. The images in the CSTD were of different sizes, and some were taken by camera from the driver’s cab across the windshield, so the reflection of the windshield had a negative effect on the quality of the picture. The AUC values of the two PR curves were 99.33% and 97.96%, respectively, and the results implied that the detection module alone was a good detector:

(10) $$Precision=\frac{true\;\;positives\;\;detected}{all\;\;detections}\times 100\%\;$$

### 6.3. Traffic Sign Classification

In the detection stage, the traffic signs were classified into two super classes: Circular and triangular traffic signs. In the classification stage, we trained three CNNs for two classification methods. One method trained two CNNs for circular and triangular traffic signs independently. The other method trained one CNN for the overall traffic sign classification. Each of the three CNNs had two convolutional layers, and each of the convolutional layers were followed by a subsampling layer. They all used a fully connected layer to produce the final classification result. The eight Gabor features of each traffic sign were used as inputs of the three CNNs, with a fixed size of 32 × 32. The first convolutional layer extracted six features for each input with 8 × 6 kernels (size 5 × 5). Additionally, the second convolutional layer extracted 12 features for each input; hence, the second convolutional layer consisted of 6 × 12 kernels with a size of 5 × 5. The 12 feature maps from the second layer were used as feature vector inputs to the fully connected layer, to produce the final classification result. The detailed structures of the three CNNs are shown in Table 6.

The input of the neural network needs to be a fixed size, but during data gathering of the traffic signs, the image sizes of the GTSRB varied from “15 × 15” to “223 × 193”, for the varying distances and angles between the camera mounted on a vehicle and the traffic sign. To solve this problem, we resized the images of the dataset to “32 × 32”, before convoluting them using the simplified Gabor filters. In this way, it was ensured that the input of the CNN was fixed to “32 × 32”.

To show the performance of our classification method, we tested the three structures of the CNNs on the dataset of the GTSRB. For training and evaluation, the GTSRB dataset was separated into training datasets and test datasets. For triangular traffic sign recognition in the CNN, we used 8970 images for training and 2790 images for testing, with a total of 15 traffic sign classes. For circular traffic sign recognition in the CNN, we used 22,949 images for training and 7440 images for testing, with a total of 20 traffic sign classes. Finally, for overall traffic recognition in the CNN, we used 39,209 images for training and 12,630 images for testing. For the three CNNs, we trained them for 500 epochs, on the GTSRB dataset with a batch size of 100, respectively.

**Figure 13:**
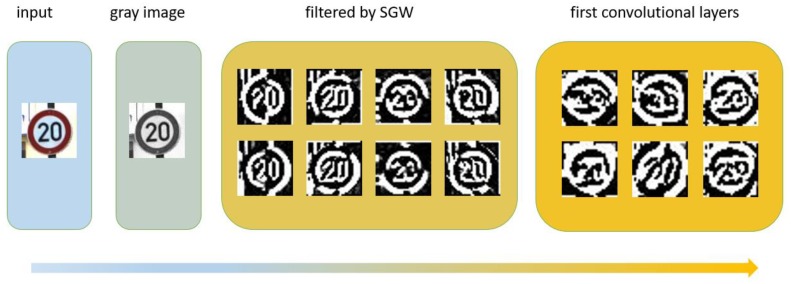
Visualization of the traffic sign transformation and the first convolution layers of the CNN.

**Figure 14:**
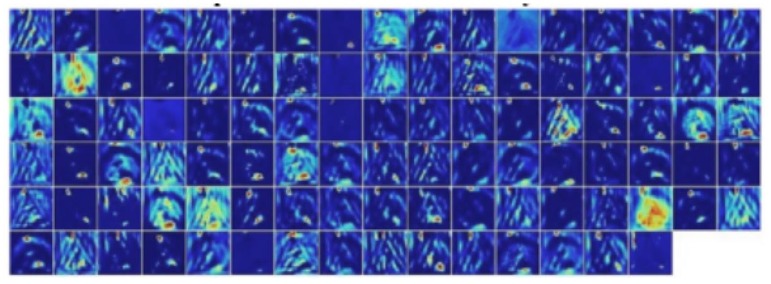
Visualization of the transformation and the first convolution layers of Reference [41].

Figure 13, from left to right, illustrates the RGB image, grayscale image, the output of the grayscale image filtered by SGW, and the visualization output of the first convolution layers of the CNN. Figure 14 shows the result of the output of the first convolution layers of the method proposed by Reference [41]. Compared with Reference [41], in the first convolutional layers of our CNNs, although the structure of the image was disrupted, similar to the first level of other CNNs, including those in Reference [41], the edge information in our CNNs was more significant. As mentioned above, the edges were the key information of the traffic signs, so this may be one of the reasons why our algorithm was more effective. This implied that edge enhancement image preprocessing technology was helpful to improve edge-based target recognition, such as traffic signs, fingerprint recognition, iris recognition, etc.

**Figure 15:**
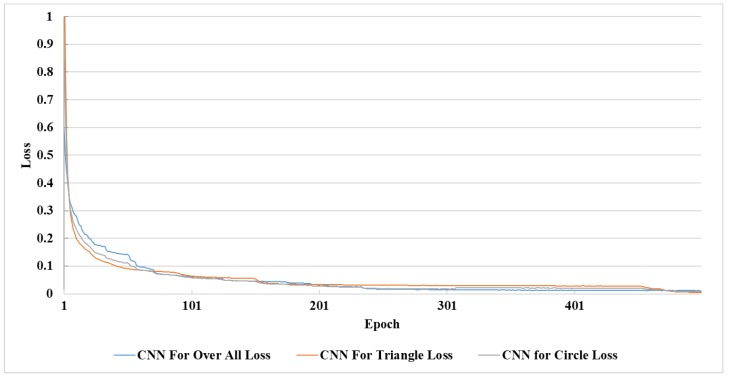
The relationship between the training epochs and training loss of triangle, circle, and overall classifications.

**Figure 16:**
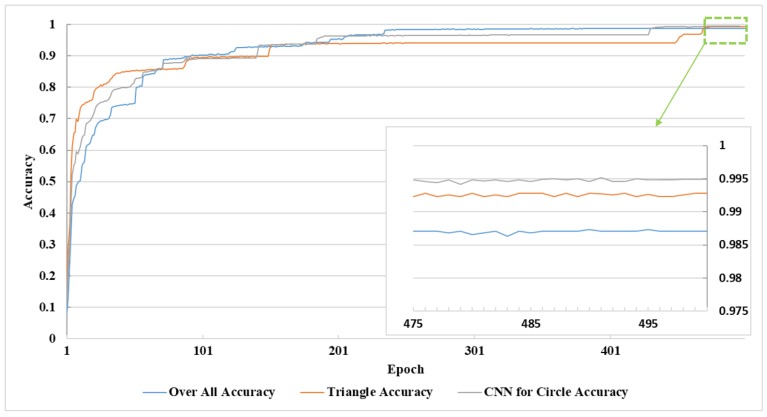
The relationship between the training epochs and classification accuracy of triangle, circle, and overall classifications.

The relationship between the loss and trained epochs of the three networks is shown in Figure 15, and the accuracy–epoch curves are shown in Figure 16. The recognition accuracy maintained a substantial increase, and the loss showed an obvious decline as the number of epochs increased due to the training before the first 90 epochs, so accuracy was below 90%. This was because the CNNs had a severe lack of training, so almost all the training samples provided a positive range for the cost function. The increase in the growth of accuracy began to decline between 90 epochs and 230 epochs and was stable between 230 epochs and 430 epochs. After 470 epochs, the recognition rate of the three networks remained at a stable value, and the value of the loss of the three CNNs was close to zero. Therefore, we concluded that under our algorithm and the current training database, the maximum recognition rate was reached after training 470 epochs with a batch size of 100.

As shown in Table 7, the accuracies were 99.28%, 99.49%, and 98.70%, for the triangle, circle, and overall classifications, respectively. Our method reached an accuracy of 99.43%, when we classified the two super classes as a whole using the two CNNs. What needs to be mentioned is that the accuracy of the CNN for circular traffic signs obtained the highest value because the amount of circular traffic signs was large, and the number of classes was small. Therefore, building a more large-scale classification database is crucial to the improvement of the traffic sign recognition rate. The detailed information of the last 25 epochs in the green dotted line box in the top right corner of Figure 16 was magnified to the inside figure, which the green arrow points to in the lower right corner.

**Figure 17:**
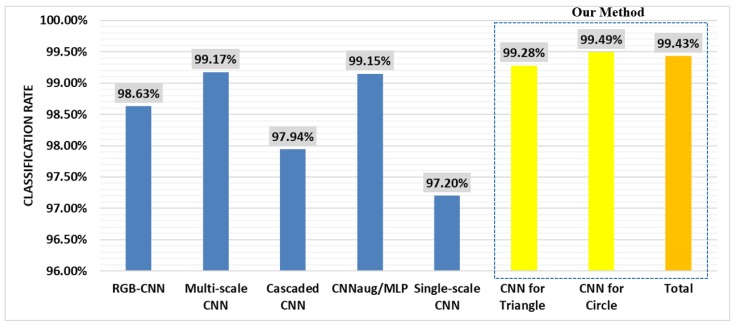
The comparison of the classification rate of ours and the state-of-the-art methods.

In Figure 17, the classification rates of our method and the other-state-of-the-art deep learning methods based on CNN are displayed, including RGB-CNN, multi-scale CNN [42], cascaded CNN [43], CNNaug/MLP [34], and single-scale CNN [15]. All the above methods were based on the dataset of GTSRB. Our method achieved superior performance as per the data shown. For further comparison, we designed an RGB-CNN. The architecture of the RGB-CNN was the same as the CNNs that we proposed in our method, except for the input, which was three matrices of red, yellow, and blue. The classification rate of the RGB-CNN, where its input was the RGB image, was 98.63%. As the only difference between our CNNs and RGB-CNN were the input features, the results suggested that our feature extractor was discriminative enough and could be used for any other intelligent classification algorithm as the feature input.

**Figure 18:**
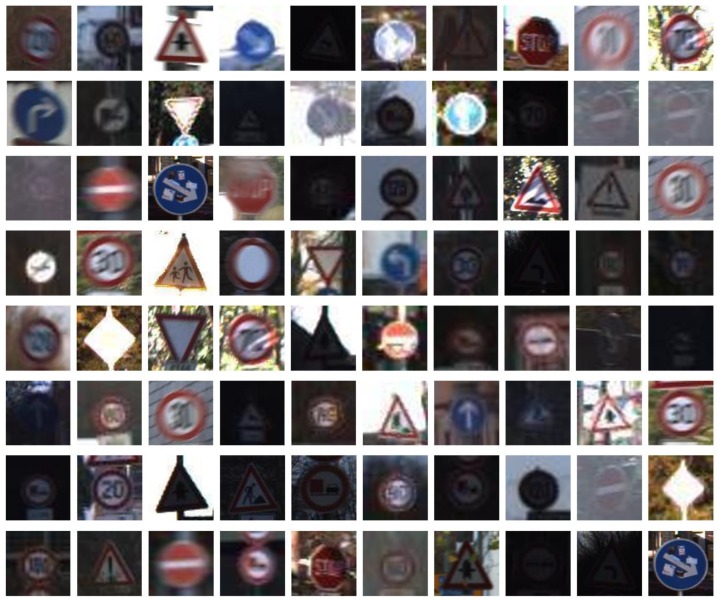
Some misclassification of the test samples.

As shown in Table 7, there were 25, 61, and 163 misrecognized traffic signs in the methods of CNN for the triangle, circle, and overall, respectively. Some misclassification samples are shown in Figure 18. The main reasons for classification errors were because the size of the traffic signs was too small, the occurrence of severe occlusion, interference from the environment by shadows, and motion blur. Some error samples could not even be identified by human beings.

The average processing time of our system included SGW filtering, region proposal, and super class classification by SVM, including preprocessing the HOG feature extraction, and finally, the classification of traffic signs. Details of the above are shown in Table 8. Through the timesaving SGW feature extraction, the processing times of this stage were reduced to 17 and 15 milliseconds, for the GTSDB and CSTD, respectively. The HOG feature extraction time stayed at 93 and 79 for the two databases, as shown in Table 8, through parameter optimization. As mentioned above, in real-time applications, the features used by the classification are shared from the detection stage, so double feature extraction is avoided, saving processing time. The average processing time of the whole process is 159 milliseconds, and about 6.3 frames per second. Our method met the needs of real-time processing.

**Figure 19:**
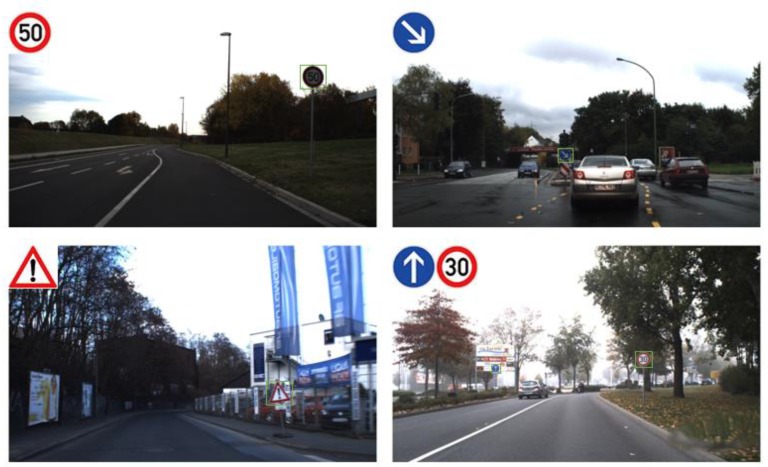
Examples of the detection and recognition results.

Figure 19 shows the results of traffic sign recognition under different road conditions using our method. The four images in Figure 19 were successfully detected and recognized, including prohibitory, danger, and mandatory traffic signs, and multiple traffic signs in one image.

## 7. Conclusions and Future Works

In this paper, a novel traffic sign detection and recognition method was presented, which aimed to address the problem of real time traffic sign detection and classification. To achieve this goal, we proposed a faster method for traffic detection and recognition. The first stage was to segment the image into ROIs by MSERs, on a simplified Gabor filter feature map. Then, we filtered out several negative samples based on defined filter rules, such as size and aspect ratio, etc. In the second stage, circular and triangular shapes were classified by the SVM on the HOG feature, and we obtained the super class to which the traffic signs belonged. In the recognition stage, the traffic signs were classified by the CNN with eight channel inputs of simplified Gabor filter maps with high accuracy. Our method completed the whole process of detection and classification at a speed of close to 6.3 frames per second, with an image size of 1360 × 800, on a regular notebook computer, with an accuracy of 99.43%. Therefore, our method is feasible for use in real time traffic sign detection and classification.

In the future, the recognition phase can be sped up using dimension reduction of feature vectors. The number and parameters of Gabor filters will greatly influence the results of the classification results. It is necessary to research the optimization of Gabor parameters using optimization algorithms, to improve the efficiency and accuracy of traffic sign detection and classification. Finally, we accelerated our method by a GPU and further improved the efficiency.

## Figures and Tables

**Table 1:** Comparison of the computational complexities of edge-detection algorithms of the Canny, traditional Gabor wavelet (TGW), and simplified Gabor wavelet SGW algorithms [30].

Algorithms	No. of Additions	No. of Multiplications
Canny	$40N^{2}$	$17N^{2}$
TGW	$48N^{2}\log_{2}N^{2}+16N^{2}$	$32N^{2}\log_{2}N^{2}+32N^{2}$
SGW	$18N^{2}$	$16N^{2}$

**Table 2:** Filter rules defined in our method.

Dataset	Parameters	Minimum	Maximum
GTSDB	Height (H)	16	128
	Width (W)	16	128
	MSERs Area/Bounding Box Area ($M_{B}$)	0.4	0.8
	Aspect ratio ($A_{r}$)	0.5	2.1
CTSD	Height (H)	26	560
	Width (W)	26	580
	MSERs Area/Bounding Box Area ($M_{B}$)	0.4	0.8
	Aspect ratio ($A_{r}$)	0.4	2.2

**Table 3:** Comparison of the recall, false negatives (FNs) between grayscale + MSERs, SGW Map + MSERs, and Reference [10].

		GTSDB	CTSD
Paper [10]	Average number of proposals	325	200
	Recall, FNs	99.63%, 1	99.44%, 3
	Time (ms)	67	90
Grayscale + MSERs	Average number of proposals	388	321
	Filtered by rules	118	99
	Recall, FNs	97.1%, 8	98.12%, 10
	Time (ms)	40	38
SGW Map + MSERs	Average number of proposals	276	178
	Filtered by rules	83	56
	Recall, FNs	99.63%, 1	99.62%, 2
	Time (ms)	46	41

**Table 4:** Different parameters of the Histogram of oriented gradient (HOG) features selected in our method.

No.	Size	Cell	Block	Stride	Bin	Gradient Direction	Dimension
HOG1	24 × 24	6 × 6	12 × 12	6 × 6	9	(0,2π)	324
HOG2	36 × 36	6 × 6	12 × 12	6 × 6	9	(0,2π)	900
HOG3	42 × 42	6 × 6	12 × 12	6 × 6	9	(0,2π)	1296
HOG4	56 × 56	6 × 6	12 × 12	6 × 6	9	(0,2π)	1296
HOG5	64 × 64	6 × 6	12 × 12	6 × 6	9	(0,2π)	1764

**Table 5:** Performance of the support vector machine (SVM) classification with different HOG features.

Data Set	Method	Detection Rate/%	Average Detection Time/MS
GTSDB	HOG1 + SVM	95.88	69
	HOG2 + SVM	99.33	93
	HOG3 + SVM	95.49	101
	HOG4 + SVM	83.25	71
	HOG5 + SVM	82.26	89
CTSD	HOG1 + SVM	94.63	62
	HOG2 + SVM	97.96	79
	HOG3 + SVM	94.08	95
	HOG4 + SVM	81.86	65
	HOG5 + SVM	81.03	78

**Table 6:** Details of the proposed CNNs architecture in our method.

Layers	CNN for Circular Traffic Signs	CNN for Triangle Traffic Signs	CNN for Overall Traffic Signs
CONV_Lay_1	Kernels: 5 × 5 × 6 × 8 Stride: 1	Kernels: 5 × 5 × 6 × 8 Stride: 1	Kernels: 5 × 5 × 6 × 8 Stride: 1
ReLU_Lay_1	Rectified Linear Unit	Rectified Linear Unit	Rectified Linear Unit
POOL_Lay_1	Method: max pooling Size: 2 × 2 Stride: 2	Method: max pooling Size: 2 × 2 Stride: 2	Method: max pooling Size: 2 × 2 Stride: 2
CONV_Lay_2	Filters: 5 × 5 × 12 × 6 Stride: 1	Filters: 5 × 5 × 12 × 6 Stride: 1	Filters: 5 × 5 × 12 × 6 Stride: 1
ReLU_Lay_2	Rectified Linear Unit	Rectified Linear Unit	Rectified Linear Unit
POOL_Lay_2	Method: max pooling Size: 2 × 2 Stride: 2	Method: Max pooling Size: 2 × 2 Stride: 2	Method: Max pooling Size: 2 × 2 Stride: 2
CONV_Lay_4 (FC)	Filters: 432 × 20 Stride: 1	Filters: 432 × 15 Stride: 1	Filters: 432 × 43 Stride: 1

**Table 7:** Accuracy of the three CNNs.

	Triangle	Circle	Overall
Training samples	8970	22,949	39,209
Test samples	2790	7440	12,630
Class number	15	20	43
Misclassification	25	61	163
Accuracy (%)	99.28	99.49	98.71
Proposed method Accuracy	99.43		-

**Table 8:** Process time of every stage of our method.

Steps	GTSDB Average Processing Time (ms/Frame)	CSTD Average Processing Time (ms/Frame)
Simplified Gabor Filter	17	15
MSERs	29	26
HOG	93	79
SVM	15	13
Classification	5	--
Total processing time	159	--
